# Evolution of a Biosynthetic Temporary Skin Substitute: A Preliminary Study

**Published:** 2015-07-20

**Authors:** Aubrey Woodroof, Richard Phipps, Collynn Woeller, George Rodeheaver, Gail K. Naughton, Emmett Piney, William Hickerson, Ludwik Branski, James H. Holmes

**Affiliations:** ^a^PermeaDerm, Inc, Carlsbad, Calif; ^b^University of Rochester School of Medicine and Dentistry, Rochester, NY; ^c^Department of Plastic Surgery at the University of Virginia, Charlottesville, VA; ^d^Histogen, Inc, San Diego, Calif; ^e^Firefighters/Regional Burn Center, Memphis, Tenn; ^f^University of Texas Medical Branch, Galveston, TX; ^g^WFBMC Burn Center, Winston-Salem, NC

**Keywords:** temporary skin substitute, wound healing, scarring, mesenchymal stem cells, variable porosity

## Abstract

**Objective:** To compare PermeaDerm to first temporary biosynthetic skin substitute (Biobrane, cleared by the Food and Drug Administration in 1979). **Methods:** Different temporary skin substitutes (Biobrane, PermeaDerm, and PermeaDerm derivatives) were tested for physical differences, impact on healing wounds, inflammatory response, and ability to allow adequate growth of dermal fibroblasts and mesenchymal stem cells without accumulation of excessive scar-forming myofibroblasts. Proliferation of fibroblasts and stem cells on various skin substitutes was measured, and myofibroblast marker accumulation was evaluated by the expression of α-smooth muscle actin and fibronectin. Fibroblast migration was measured by tracking viable cells with MTT [3-(4,5-dimethylthiazol-2-yl)-2,5-diphenyltetrazolium bromide] dye. **Results:** In vivo testing shows PermeaDerm works well as a temporary skin substitute, performing better than Biobrane with respect to inflammation and fluid accumulation. Tissue culture techniques revealed that cells on PermeaDerm grow in a more uniform fashion and migrated to a greater extent than cells on Biobrane. Furthermore, cells grown in the presence of PermeaDerm expressed lower levels of the myofibroblast markers α-smooth muscle actin and fibronectin than cells grown on Biobrane. **Conclusion:** PermeaDerm with variable porosity possesses all attributes and properties known to be important for a successful temporary skin substitute and enables the clinician to control porosity from essentially zero to what the wound requires. The ability of the clinician to minimize wound desiccation without fluid accumulation is related to the reduction of punctate scarring.

Early surgical excision of major burns has been a key factor in reducing mortality and morbidity.^1^^-^4 Once the excision is performed, the challenge is to close the excised area with an effective skin substitute until autografting. Biobrane was found equally effective as frozen cadaver allograft^5^ for this purpose. Other temporary skin substitutes^6^^-^12 such as pig skin and human amniotic membrane have been used for temporary protection of the excised area, although not considered as effective as frozen cadaver skin (reference criterion). Methodology^13^ was developed to comparatively measure and quantify adherence of skin substitutes to deepithialized surfaces, as well as characterizing the mechanism of the acute and secondary adherence processes. The attributes and properties of an ideal temporary skin substitute have been identified,^12^,^14^^-^18 and the search for an ideal skin substitute continues. Use of a biosynthetic skin substitute has been shown effective on second-degree pediatric burns.^19^^,^^20^ Open holes in a biosynthetic skin substitute cause punctate scarring on scald burns.^21^

To surgeons expert in the art of excision, a skin substitute is something that protects viable tissue until autografting and a “take” has occurred. Others^22^ use the terms “smart dressing,” “sophisticated dressing,” or “bioengineered alternative tissue” for materials that provide attributes/properties of a temporary skin substitute.

Improvement over Biobrane was introduced in 2008 as AWBAT,^17^^,^^23^ and preliminary clinical studies showed a low infection complication. Another improvement in biological coating of 3-dimensional (3D) matrix, AWBAT Plus^24^ was introduced in 2010 and shown in vitro to stimulate fibroblast growth, collagen synthesis, and α-smooth muscle production.

Chronic or slow-healing wounds also require excision and/or debridement of necrotic tissue and often closure with a skin substitute. The primary objective is protection of viable tissue and minimization of infectious complications. This objective can be achieved with an adherent “smart dressing” or “bioengineered alternative tissue” that minimizes fluid accumulation beneath the primary dressing, minimizes dressing changes and pain, and reduces the cost of care.

The work described herein began in the early 1970s^13^^,^^16^ and continues toward the objective of finding better ways of closing the debrided/excised wound, improving safety, and lowering the cost of care. It may be possible to advance the art of wound closure by adding relatively stable components to the biological coating of the 3D structure of PermeaDerm, for example, an antiscar compound and/or a mixture of native collagen with high molecular weight (eg, 300,000, 600,000, and 900,000 Da).

## METHODS

Several new temporary skin substitute prototypes were evaluated (Table 1): PermeaDerm B and CW vary in the number of slits per unit area; PermeaDerm T contains high-molecular-weight collagen (10% bovine tropocollagen and 90% standard biologicals, which are a mixture of porcine gelatin/aloe); PermeaDerm AS contains an antiscar agent in the silicone, whereas PermeaDerm AS-2 contains the antiscar compound, which is soluble in equal volumes of ethyl alcohol and dimethylsulfoxide, in both the silicone and the biological coating (gelatin/aloe) of the 3D matrix. Physical testing was performed with PermeaDerm B or CW; neither prototype contained biological coatings. Sterilization was performed by exposing each prototype to 25 kGy of electron-beam irradiation. Safety and efficacy testing of prototypes after sterilization are described herein.

Physical testing done at Manufacturing Solutions Center (MSC) on PermeaDerm B or CW (without biological coating) versus Biobrane: (1) moisture vapor control—Water Vapor Transmission (WVT) ASTM Standard E96, 2000e1; (2) water flow rate through membranes; and (3) breaking force and elongation ASTM D5035, 2011.

Analytical chemistry (ECA, San Diego, CA) was performed to characterize the amount of collagen (hydroxyproline) and *Aloe vera* (Immuno-10) present in biological coatings. Scanning electron microscopy of Biobrane versus PermeaDerm relaxed and with stretch was performed (S & N Labs, Santa Ana, CA).

### In vivo comparative testing of Biobrane versus PermeaDerm

The topical tissue response and potential systemic effects of PermeaDerm (B slit pattern with standard biological coating of gelatin/aloe) were compared with Biobrane in a 2-week toxicity study involving 24 Sprague-Dawley rats (NAMSA, Northwood, OH). Approximately 2 × 2-cm full-thickness dermal wounds were created on each side of the back (2 wounds per animal). All wounds were covered with either the test article, PermeaDerm, or the control, Biobrane. On day 14, the animals were killed humanely, examined for gross abnormalities externally and internally, and each wound site was excised in toto, including approximately 1 cm of marginal skin surrounding the wound sites. The sites were fixed flat in 10% neutral buffered formalin. The liver, spleen, both kidneys, submandibular lymph node, both adrenals, heart, thymus, mesenteric lymph node, lung, and both testes or ovaries were collected from each rat and fixed in 10% neutral buffered formalin. Each wound was trimmed to obtain a central strip of tissue. All tissues were processed to obtain tissue sections that were stained with hematoxylin and eosin for light microscopic examination. Additional sections of the wound sites were prepared and stained with Masson's trichrome stain. All wound sections were evaluated for crust formation, epidermal hyperplasia, reepithelialization, maturity of granulation tissue, presence of necrosis (separate from pockets of inflammation), inflammation, presence of foreign material (eg, dressing fragments), and hemorrhage.

A second in vivo model evaluated the effectiveness of PermeaDerm prototypes (B slit pattern with standard biological coating or standard coating with small amount of high-molecular-weight native collagen) compared with Biobrane in wound healing used a New Zealand White rabbit skin injury model. Both surgery and animal care were conducted in accordance with a protocol approved by the University of Virginia Animal Care and Use Committee. After anesthesia, the hair on the dorsum was removed with electric shears and then depilated. The animal was draped and the skin prepared with 3 repeated applications of iodophor antiseptic solution, followed by alcohol removal. Once the skin was prepped, a 5 × 10-cm rectangle was marked on each side of the spine, midway between the scapulae and the pelvis. Using aseptic technique and a Brown dermatome, a 0.015-in thickness of the skin was removed from the designated site. Punctate bleeding was stopped with sterile gauze and pressure. Each donor site received 5 test strips, 1.5 × 4.3 cm. Each test strip was oriented so that the long axis of the strip was perpendicular to the spine. The distal end of each strip was secured to the wound bed with a single suture. Each 4.3-cm long test strip was elongated to 4.5 cm (4.6% elongation) and secured with a suture. This elongation slightly opened the pores or slits in the test strips. Each donor site was dressed with an absorptive pad (Exu-Dry; Smith & Nephew, Inc, St Petersburg, Fla), wrapped circumferentially with a breathable adhesive film, and followed by a compressive wrap. Each donor site received 5 test strips for a total of 10 strips. Of the 10 strips, 2 were Biobrane (B), 4 were PermeaDerm (P = PermeaDerm B), and 4 were PermeaDerm T (PT = PermeaDerm B with native collagen). The strips were randomized for placement, with each donor site receiving 1B, 2P, and 2PT strips. Dressing assessment was performed at 4, 7, 11, and 13 days. On day 13, the rabbit was anesthetized and the dressings removed. On days of analysis, each test strip was assessed for adherence, accumulation of fluid beneath the skin substitute, fluid accumulation in secondary dressing (Exu-Dry), and degree of reepithelialization.

### In vitro comparative testing of PermeaDerm and Biobrane

The effects of various temporary skin substitutes on fibroblast and mesenchymal stem cell (MSC) growth, proliferation, and myofibroblast differentiation were tested. The membranes used were as follows: Biobrane; PermeaDerm; PermeaDerm T (T = PermeaDerm B with high-molecular-weight native collagen); PermeaDerm + antiscar agent placed in the silicone layer (PermeaDerm AS); and PermeaDerm + antiscar agent placed in both the silicone layer and the biological layer (PermeaDerm AS-2). The antiscar compound used in this study is the small molecular ionophore, salinomycin.^25^ The impact of each temporary skin substitute was evaluated to monitor cell proliferation, cell morphology, and myofibroblast marker expression on human dermal fibroblasts (DFs) and human MSCs.

#### Proliferation

DFs or MSCs were seeded into 48-well plates with MEM medium containing 0.1% to 10% fetal bovine serum and a 0.8-cm^2^ strip of specified skin substitute for up to 7 days. To assess cell morphology, images of cells grown on the temporary skin substitutes were captured using an Olympus microscope and a digital camera. The ^3^[H]thymidine assay was used to measure the relative rate of human DF and MSC proliferation in the presence of specific skin substitutes. Exogenous ^3^[H]thymidine (Perkin Elmer, Waltham, Mass) was added to each well so that it can be incorporated into newly synthesized DNA in proliferating cells. Cells were cultured in low serum concentration (0.1%) to render them moderately quiescent. Addition of 10% serum was used to stimulate proliferation and myofibroblast formation. Measurement of myofibroblast markers was performed by quantitative real-time PCR: total RNA was isolated using the RNeasy kit (Qiagen, Valencia, Calif) following the manufacturer's protocol, treated with DNase (Qiagen), and assessed for purity using spectroscopy. One hundred nanograms of total RNA was used with iSCRIPT reverse transcriptase (BioRad, Hercules, Calif) to generate cDNA. Real-time PCR reactions were performed using SYBR green reagent. Relative gene expression was normalized to reference gene expression (7S RNA) as previously described. Primers used in this study are as follows: α-smooth muscle actin (αSMA) fwd: (CCCACAATGTCCCCATCTATG), αSMA rev: (AGTTTCTCCTTGATGTCC CG); fibronectin (FN1) fwd: (CCACTTCCCCTTCCTATACAAC), FN1 rev: (ACTGAT CTCCAATGCGGTAC); 7S RNA: 7S fwd: (ACCACCAGGTTGCCTAAGGA), 7S rev (CACGGGAGTTTTGACCTGCT).

As another approach to measure fibroblast attachment, migration, and proliferation on temporary skin substitutes, human neonatal fibroblasts were grown directly on Biobrane, PermeaDerm, or PermeaDerm T. Membranes (skin substitutes) were cut to standard size 15-mm disc samples placed in 15-mm polystyrene wells with 3D surface facing up. Cells were seeded after trypsinization to disperse on membranes and then incubated with DMEM medium containing 10% calf serum. After various incubation times, culture wells were treated with MTT (3-(4,5dimethylthiazol-2-yl)-2,5-diphenyltetrazolium bromide) dye, which forms a purple precipitate in the presence of viable cells due to their ability to reduce MTT by cellular oxidoreductase enzymes. On the basis of the presence of the reduced purple precipitate, endpoints measurements were cell adhesion, cell proliferation, and cell migration. Adhesion was measured in short-term (24- to 49-hour) cultures. Proliferation and migration assays were performed at days 7 and 21 to measure cell growth and response to different biological coatings on the skin substitutes. Migration studies used photo endpoints to compare migration from the initial seeding space to the space of cells upon termination of experiment.

## RESULTS

### Physical and chemical testing of temporary skin substitutes

**Figure 1:**
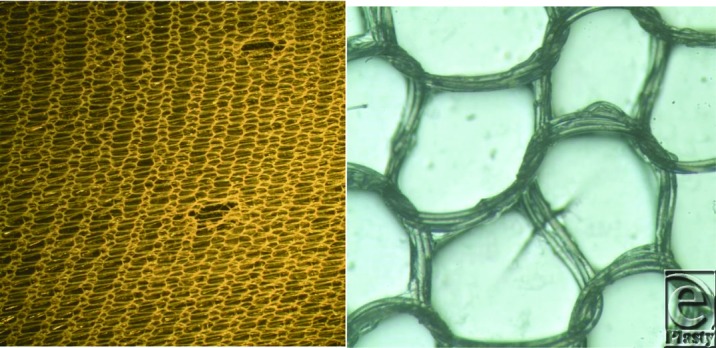
Photographs of Biobrane. Left, Holes. Right, Triple stranded nylon knitted component. Structure similar to 1980 Biobrane product,^15^ where acute adherence (5 hours) and secondary adherence (72 hours) to excised rat wounds were quantitatively determined. Acute adherence (about same as pigskin and allograft) was 50 g/cm^2^; secondary adherence was more than 200 g/cm^2^ (much greater than pigskin and allograft, which were about 150 g/cm^2^). Objective of engineering design for AWBAT, AWBAT Plus, and PermeaDerm was to decrease secondary adherence, as compared with Biobrane, to a value close to that of a biological dressing (150 g/cm^2^). Holes in Biobrane never close; however, they will enlarge if stretched.

**Figure 2:**
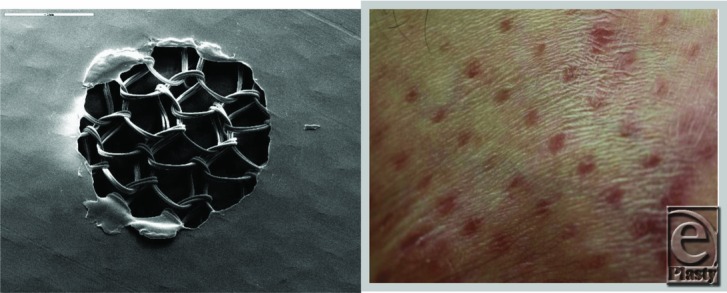
AWBAT. Left, Silicone side facing up; double strand knitted nylon below the hole can be seen. The hole never closes; diameter of the hole can be increased with elongation/stretching. Right, Example of punctate scarring^20^ associated scar with open pores of Biobrane.

**Figure 3:**
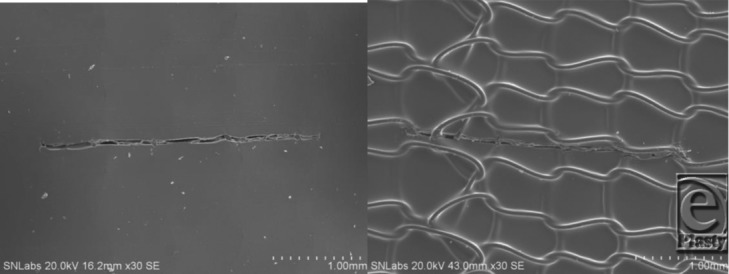
Left, Scanning electron microscopy of PermeaDerm B (burns) silicone surface, which contains 2280 slits/sq ft; each slit is 0.250 in long. Illustrates the presence of a single slit; no stretch. Therefore, the slit is closed, providing minimum wound desiccation, which is known to cause punctate scarring. Right, PermeaDerm B view from 3-dimensionnal nylon side of PermeaDerm B. Illustrates slit presence and closed when no stretch applied by the clinician or stretch applied parallel to the direction of the slit.

**Figure 4:**
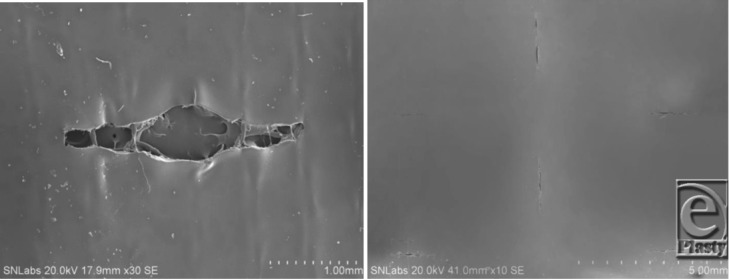
Left, Scanning electron microscopy of PermeaDerm B.^17^ View from silicone surface under slight stretch. Illustrates increase of slit opening, which facilitates movement of exudate from wound surface through PermeaDerm B into sterile absorbent secondary dressing. Secondary dressing can be replaced without interruption of the healing process. When stretched perpendicular to direction of slit, the slit opens. Right, PermeaDerm CW. Illustrates 6 slits, 2 vertical and 4 horizontal, as seen from silicone surface. There are 4464 vertical and horizontal slits per sq ft; each slit is 0.180 in long in PermeaDerm CW, and this design possesses maximum conformance/stretchability to adapt to irregular wound surfaces. Currently, the slits are in the closed position; when stretched in any direction, slits open proportional to the degree of elongation.

**Figure 5:**
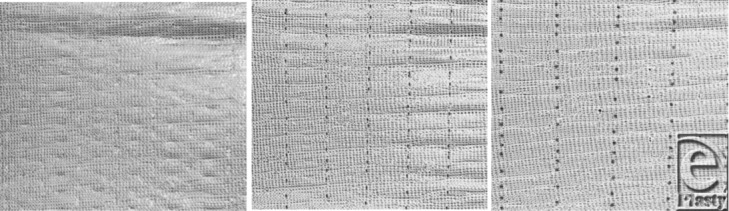
PermeaDerm B. Left, No elongation. Middle, Slight elongation. Right, Full elongation.

A list of all temporary skin substitutes is given in Table 1 (Figs 1–5). All physical testing was performed on slitted silicone/nylon sheets (2 patterns used—B or CW) of PermeaDerm prototypes without biological coating. Biobrane WVT rates were higher than PermeaDerm B or CW rates. Fluid transfer measurements showed PermeaDerm prototypes to be essentially zero without stretch and about 50% of the Biobrane rate with stretch from 3.1 to 4 cm. Biobrane required more force to break than PermeaDerm prototypes, although all had more than adequate strength and elongation to perform as an ideal temporary skin substitute. Chemical testing showed aloe to be present in PermeaDerm prototypes and absent in Biobrane; collagen (hydroxyproline) was present in PermeaDerm prototypes as well as Biobrane (Table 2).

WVT ASTM Standard E96, 2000e1: By MSC, Conover, NC. Three samples of each material:

- Biobrane: 1055 g/m^2^/24 h; SD = 45.6
- PermeaDerm CW: 834.9 g/m^2^/24 h; SD = 58.4
- PermeaDerm B: 870 g/m^2^/24 h; SD = 101.4

Fluid (Water) Transfer: By MSC, Conover, NC. Three samples of each material:

- Biobrane: 19.412 g flowed in 40 to 40.3 seconds.
- PermeaDerm CW with no stretch: No flow, essentially zero porosity. With stretch from 3.1 to 4 cm; about 20 g flowed in 15 to 20 seconds.
- PermeaDerm B with no stretch: No flow, essentially zero porosity. With stretch from 3.1 to 4 cm; flow complete in about 23 seconds.

Breaking Force and Elongation ASTM D5035, 2011: By MSC, Conover, NC. Test performed in 2 directions:

- Biobrane: Breaking force 5737.4 g, 2104.3 g; elongation 181%, 182%
- PermeaDerm CW: Breaking force 1178.4 g, 4863.8 g; elongation 126%, 252%
- PermeaDerm B: Breaking force 3221.8 g, 2012.3 g; elongation 243%, 120%

### Biological testing of temporary skin substitutes

#### In vivo evaluation of PermeaDerm compared with Biobrane in animal models of wound healing

**Figure 6:**
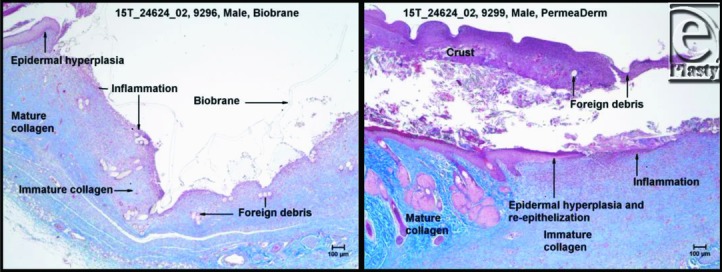
Masson's trichrome staining of Biobrane site of a male (left). Biobrane is overlying the wound and is present as numerous aggregates of foreign debris within the wound. Masson's trichrome staining PermeaDerm site of a male. The amount of foreign debris is scant and a slight amount of reepithelialization is present.

The topical tissue response and potential systemic effects of PermeaDerm were compared with Biobrane in a 2-week toxicity study involving 24 Sprague-Dawley rats (NAMSA) as described in the “Methods” section. After the study, tissues were analyzed and scored as described earlier. There were no clinical pathological, gross pathological, or histopathological evidence of toxicity (Tables 3–5 and Fig 6). The wounds of all animals appeared to be at the same stage of healing by granulation tissue (Fig 6). There were no areas of necrosis, mineralization, or fibrosis within the wounds. The average scores of the test wound sites were generally similar to the average scores of the control sites for the presence of crust, epidermal hyperplasia, reepithelialization, and hemorrhage. The exceptions were inflammation and presence of foreign debris (Table 3 and Fig 6). The average score for inflammation of female test (PermeaDerm) sites (7.0) was similar to, but slightly lower than, the average score of inflammation for the control (Biobrane) sites of females (7.6) (Table 3). For males, the average score for inflammation at test sites (7.0) was considerably lower than the average score for the male control sites (9.7). The probable cause of the inflammation was a foreign body response with an increased number of macrophages in the male control sites. Most of the foreign debris appears to be fragments of the dressing. The average score for foreign debris was lower for the test wound sites (male wound sites [1.3] and female wound sites [0.8]) than for the control wound sites (male wound sites [2.9] and female wound sites [2.5]). The difference in the physical appearance of the dressing accounts for differences in the average scores for foreign debris.

**Figure 7:**
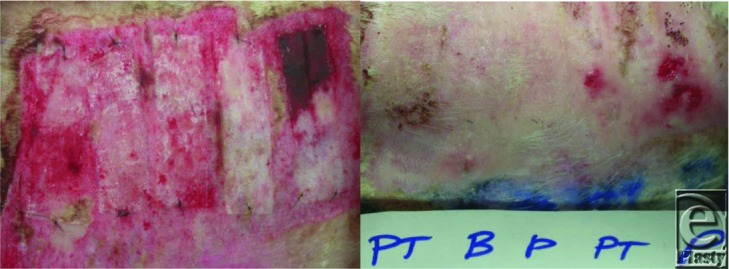
Rabbit study. Left, Photograph at 3 days, wound seems deeper on the far right. Right, Photograph at 13 days, wounds healed except for a small area on the right.

Using the skin donor site model with the New Zealand White rabbit (Fig 7), results demonstrate that PermeaDerm and Biobrane act as effective temporary skin substitutes. Briefly, analysis of various time points post–skin graft revealed the following observations: on day 0, the Biobrane (B) strips laid flatter than the PermeaDerm (P) or PermeaDerm T (PT) strips, which tended to curl up at the edges. On day 3, the overlying absorptive pads (Exu-Dry) were filled with exudate. On day 7, fluid accumulation was evident in the secondary dressings (Exu-Dry) over each test site, although considerably less than observed on day 3. No fluid was observed under any test strip (B, P, and PT), and all test strips were adherent and dry on days 3 to 13. On day 11, one of the Biobrane strips had lifted off the donor site revealing reepithelialization. All other test strips were still adherent. On day 13, all dressings had lost a significant degree of adherence due to the reepithelialization. All dressings appeared to be performing equally. The test strips were only adherent at spots of deeper wound thickness.

#### In vitro evaluation of PermeaDerm and PermeaDerm derivatives compared with Biobrane

**Figure 8:**
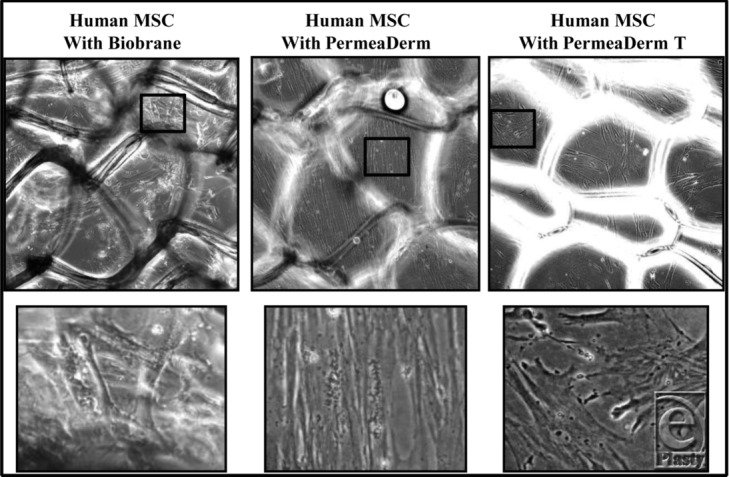
Human MSCs display enhanced wound-healing properties on PermeaDerm and PermeaDerm T compared with Biobrane. Human MSCs were cultured on membranes for 7 days in the presence of 10% fetal bovine serum before cell images were taken. Representative images of MSCs grown on various temporary skin substitutes are shown. The lower panels represent blown up images of the boxed area in the upper panels. MSCs growing on PermeaDerm and PermeaDerm T grow in a more uniform and dispersed pattern than those on Biobrane. The cells on Biobrane tend to pile up on each other forming cellular “clumps.” Original magnification 200×. MSC indicates mesenchymal stem cell.

Human MSCs or DFs were cultured in appropriate media for propagation and maintenance before seeding cells in desired culture dishes for evaluation of growth, proliferation, and myofibroblast differentiation in the presence of PermeaDerm derivatives or Biobrane. To evaluate cell morphology on various membranes, 4 × 10^5^ MSCs were plated into 24-well plates containing 1.6 cm^2^ of Biobrane, PermeaDerm, or PermeaDerm T. Membranes were anchored in place in the culture wells by the addition of a cloning ring. MSCs were allowed to attach and grow for 1 week in the respective wells before cells were analyzed by microscopy. MSCs grown in the presence of Biobrane grew in uneven cell clusters or “clumps” (Fig 8, left panel, both top and bottom images). MSCs on PermeaDerm or PermeaDerm T grew in a more uniform monolayer (Fig 8, middle and right panels).

**Figure 9:**
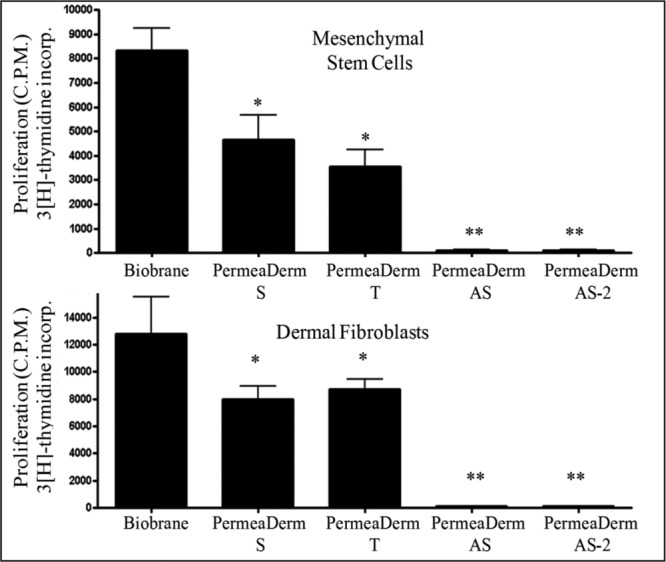
Human mesenchymal stem cells (top) and human dermal fibroblasts (bottom) grow at different rates when cultured with PermeaDerm derivatives or Biobrane. Cells were cultured with membranes (treatment) for 24 hours in 10% fetal bovine serum before the addition of 3[H]thymidine for an additional 96 hours to measure proliferation. PermeaDerm and PermeaDerm T significantly reduce proliferation compared with Biobrane. Addition of the antiscar compound, salinomycin, to PermeaDerm dramatically decreased cellular proliferation of both mesenchymal stem cells and dermal fibroblasts. Addition of antiscar compound (AS) to PermeaDerm dramatically reduced cell proliferation. Data were analyzed by analysis of variance using Prism software from Graphpad (*P < .05, **P < .01).

To test the growth and proliferation of cells on various temporary skin substitutes, 2 × 10^5^ MSCs or DFs were plated in 48-well plates that contained 0.8 cm^2^ of Biobrane, PermeaDerm, PermeaDerm T, PermeaDerm AS, or PermeaDerm AS-2. After cells attached to the membrane, proliferation was measured using the ^3^[H]thymidine incorporation assay (Fig 9).

Interestingly, growth rates on the various membranes were significantly different and Biobrane allowed for the highest level of proliferation in both MSCs and DFs, followed by PermeaDerm and PermeaDerm T. Proliferation of MSCs and DFs was almost completely prevented by PermeaDerm coated with the antiscarring compound, salinomycin. Coating of only the silicone component or coating of the silicone and biological components of PermeaDerm with salinomycin prevented proliferation. Importantly, coating of salinomycin did not result in cell death, as we have previously shown that salinomycin does not induce cell death of human fibroblasts at the doses used in these studies.^24^

**Figure 10:**
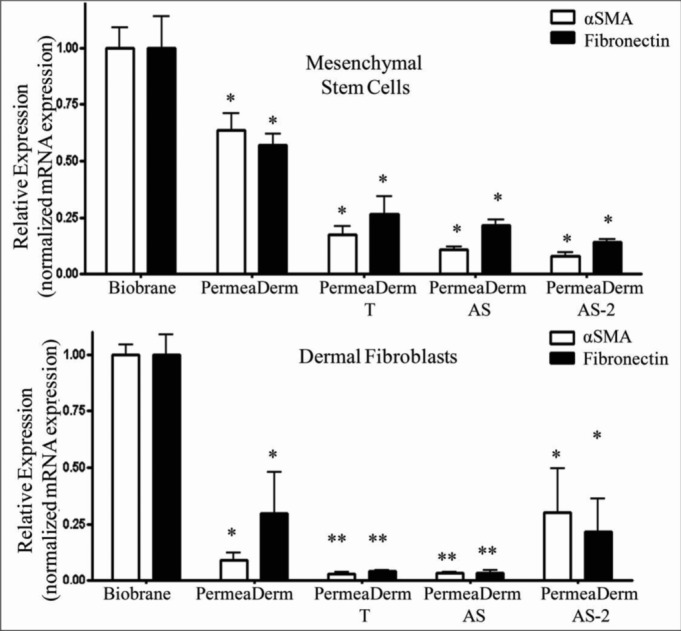
Human mesenchymal stem cells (top) and human dermal fibroblasts (bottom) express lower levels of myofibroblast markers when cultured with PermeaDerm derivatives than with Biobrane. Human mesenchymal stem cells were cultured on membranes for 7 days in the presence of 10% fetal bovine serum before total RNA was extracted and analyzed by quantitative real-time PCR for expression of the myofibroblast markers, αSMA, and fibronectin. PermeaDerm and its derivatives reduced expression of myofibroblast markers compared with cell grown with Biobrane. Addition of antiscar compound (AS) to PermeaDerm derivatives decreased myofibroblast marker production. Data were analyzed by analysis of variance using Prism software from Graphpad (*P < .05, **P < .01). αSMA indicates α-smooth muscle actin.

To evaluate the formation of myofibroblasts on various temporary skin substitutes, 4 × 10^5^ MSCs or DFs were plated in 24-well plates that contained 1.6 cm^2^ of Biobrane, PermeaDerm, PermeaDerm T, PermeaDerm AS, or PermeaDerm AS-2. MSCs or DFs were then cultured for 7 days in the presence of 10% fetal bovine serum, which allows the formation of scar-forming myofibroblasts. After treatment, total RNA was extracted from the cells and analyzed for the expression of the myofibroblast markers, αSMA, and FN1 as described in the “Methods” section (Fig 10). PermeaDerm derivatives showed a reduced level of myofibroblast marker expression compared with Biobrane. Interestingly, the addition of salinomycin to PermeaDerm dramatically inhibited myofibroblast formation in both MSCs and DFs.

**Figure 11:**
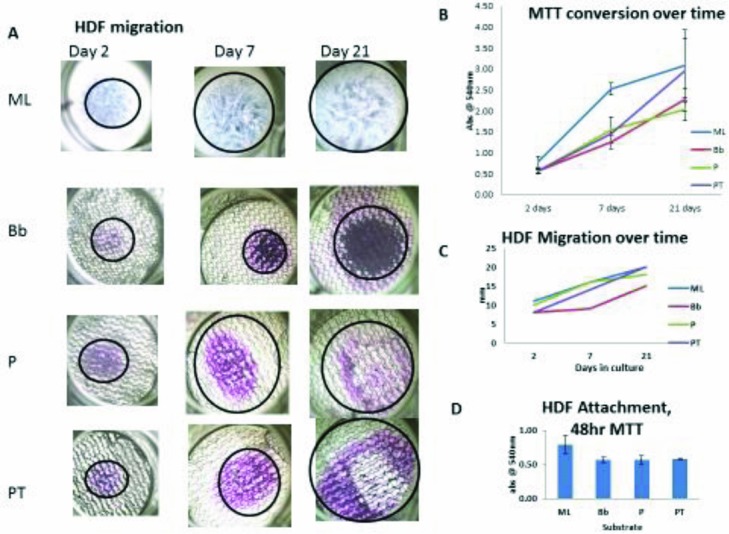
HDF migration is increased with PermeaDerm membranes compared with Biobrane. (a) 100× bright-field images of MTT-stained cells on various substrates. HDF indicates human dermal fibroblast; ML, monolayer on polystyrene; Bb, Biobrane; P, PermeaDerm B; and PT, PermeaDerm T. With time in culture, HDFs proliferate and migrate across surface. Clearly, there is variation in proliferation and migration dependent on substrate formulation. (b) The variation over time in HDF proliferation where the ML shows the most rapid attachment and proliferation whereas PT has the highest rate of proliferation throughout the culture time. (c) Quantitates the migration rate of the HDFs on the various substrates, with PT showing the greatest rate overall. (d) The 48-hour MTT conversion data serve as a measure of initial attachment. ML serves as the 100% reference point, with the other materials falling within 75% of ML binding. This indicates that there is a slight reduction in attachment potential between polystyrene and the other substrates, but this becomes irrelevant with time.

Human DF migration is increased with PermeaDerm membranes compared with Biobrane. DFs were plated on Biobrane, PermeaDerm, or PermeaDerm T and incubated for various times before the addition of MTT viability reagent as described in the “Methods” section. Cell attachment, growth, and migration were evaluated by the presence of the reduced form of MTT, which forms a purple precipitate (Fig 11). Interestingly, culture in the presence of PermeaDerm or PermeaDerm T allowed for more even distribution and migration of DFs than Biobrane.

## DISCUSSION

Biobrane has been clinically available for 36 years (the Food and Drug Administration cleared April 1979) and has been shown to be efficacious as a temporary skin substitute. Herein, we show through a series of physical, chemical, and biological tests that PermeaDerm and PermeaDerm derivatives offer a potentially major advance in the field of wound care compared with Biobrane.

Physical testing was performed on slitted silicone/nylon sheets (2 patterns used—B or CW) of PermeaDerm prototypes without biological coating. Biobrane WVT rates were higher than PermeaDerm B or PermeaDerm CW rates. Fluid transfer measurements showed PermeaDerm prototypes to be essentially zero without stretch and about 50% of the Biobrane rate with stretch from 3.1 to 4 cm. Biobrane required more force to break than PermeaDerm prototypes, although all had more than adequate strength and elongation to perform as an ideal temporary skin substitute. Chemical testing showed aloe to be present in PermeaDerm prototypes and absent in Biobrane; collagen (hydroxyproline) was present in PermeaDerm prototypes as well as Biobrane.

In vivo testing of temporary skin substitutes shows that after 14 days, healing of full-thickness dermal wounds in rats with a PermeaDerm dressing was similar to healing with a Biobrane dressing, microscopically, with exceptions. The use of the PermeaDerm dressing resulted in less foreign debris in wounds (males and females) and less inflammation (males), which were both desirable features. After 14 days, the inflammation was mainly occurring as a foreign body response to dressing material within wounds. The Biobrane dressing was more porous than the PermeaDerm dressings, thus allowing for more foreign body inflammatory reaction, a hindrance to healing. Comparison of Biobrane with PermeaDerm (Table 5) shows that PermeaDerm performance (no fluid accumulation in 24 rats) is superior to Biobrane (fluid accumulation in 7 of 24 rats).

In the rabbit model, the performance of PermeaDerm and PermeaDerm T was similar to that of Biobrane. All 3 dressings adhered to the wound bed, allowed wound exudate to escape through the membrane without any accumulation under the dressing, and did not inhibit normal healing under the dressing. Each of these dressings performed as an ideal temporary skin substitute. Wound healing rate is influenced by 3D structure of the skin substitute, biological properties and shape of the coating of the 3D surface of the skin substitute, variations in the wound (how deep and uniform the dermatome cut), as well as other skin substitute attributes/properties. Rodeheaver et al^26^ described the influence of the 3D structure of a biosynthetic skin substitute (Biobrane) on acute and secondary adherence. Greenwood^27^ clinically compared Biobrane (15/3 denier nylon) versus AWBAT (15/2 denier nylon). He found that both materials had excellent acute adherence; Biobrane separated from healed scald wounds on average at 9.2 (SD = 1.77) days and AWBAT separated at 8.7 (SD = 2) days. Differences were not statistically significant.

Furthermore, our in vitro studies using human fibroblasts and MSCs offer some important evaluations of PermeaDerm and PermeaDerm derivatives compared with Biobrane. Both human MSCs and DFs grow well on PermeaDerm and PermeaDerm with native collagen. The ability of PermeaDerm to limit excessive proliferation of cells at the open wound may be seen as a great advantage, as unwanted and excessive cell proliferation during wound healing is associated with scar formation. Scarred tissues often lose function as a result of hypercellularity and the presence of high levels of myofibroblasts, which express contractile αSMA and secrete high levels of extracellular matrix such as FN1. Cells grown in the presence of PermeaDerm and PermeaDerm with native collagen also displayed reduced expression of myofibroblast markers compared with Biobrane, further highlighting the important beneficial differences between membrane materials.

MSCs grown in the presence of PermeaDerm displayed uniform monolayer growth across the membrane, which contrasts with the growth of cells on Biobrane. Cells grown in the presence of Biobrane grew close together and even on multiple layers creating cellular clumps that may alter the cellular microenvironment of a healing wound. Cells grown in close proximity and in clusters lead to exuberant TGFβ production and unwanted cellular signaling by myofibroblasts, thus leading to further unwanted and uneven scar formation. The ability of PermeaDerm to allow increased monolayer outgrowth may limit excessive myofibroblast formation, proliferation, and signaling.

Another key and exciting finding was to demonstrate proof of principle that PermeaDerm could be coated with a small molecule inhibitor of scarring and that this coating is biologically active. For the first time, we show that PermeaDerm can be coated with salinomycin, a potent antiscarring molecule that limits myofibroblast formation, proliferation, contraction, and extracellular matrix production.^25^ Our results reveal that salinomycin may function as an antiscarring molecule when it is added directly to the PermeaDerm matrix. The ability of PermeaDerm to be coated, either in the silicone component or additionally in the biological component with an antiscarring compound, reveals the potential for further optimization of PermeaDerm formulations. A limitation of our initial findings is that, in the future, the amount and release of salinomycin or other antiscarring compounds need to be optimized to permit antiscarring activity along with minimal to no inhibitory activity of other cells such as epithelial cells.

## CONCLUSIONS

PermeaDerm with variable porosity possesses all attributes and properties known to be important for a successful temporary skin substitute and enables the clinician to control porosity from essentially zero to what the wound requires. The ability of the clinician to minimize wound desiccation is related to the reduction of punctate scarring associated with open holes in a biosynthetic temporary skin substitute.

There was no clinical pathological, gross pathological, or histopathological evidence of toxicity of PermeaDerm. The Biobrane dressing was more porous than the PermeaDerm dressings, thus allowing for more foreign body inflammatory reaction, a hindrance to healing. In 24 full-thickness rat wounds, PermeaDerm had zero fluid accumulation as compared with 7 of 24 wounds covered with Biobrane.

Animal studies (24 rats and 1 rabbit) show excellent acute adherence of Biobrane and PermeaDerm. Adherence is believed to be a critical and essential property of a skin substitute: “If it don't stick, it won't work!” (Dr Charles Baxter, Parkland Burn Center, Dallas, TX, oral communication, 1980). PermeaDerm prototypes with variable porosity contain a lighter, knitted nylon component (15/1 denier) than Biobrane (15/3 denier) and have essentially zero porosity unless stretched by the clinician.

Both human MSCs and DFs were able to grow well in a cell monolayer with PermeaDerm and PermeaDerm with native collagen (PermeaDerm T). The ability of PermeaDerm to limit aberrant cellular proliferation and myofibroblast formation suggests PermeaDerm to be superior to Biobrane in wound healing. In addition, coating PermeaDerm with an antiscarring compound such as salinomycin highlights the potential use of antiscarring agents directly in wound healing 3D matrices.

## Figures and Tables

**Table 1:** Name and description of temporary skin substitutes used in the study

Temporary skin substitute	Notes
Biobrane	Cleared by the FDA in 1979, nylon mesh with collagen
PermeaDerm B	Nylon mesh with silicone with 2280 slits/sq ft for burns
PermeaDerm CW	Nylon mesh with silicone with 4464 slits/sq ft for chronic wounds
PermeaDerm	PermeaDerm B with standard biological coating
PermeaDerm T	PermeaDerm B with standard coating and HMW native collagen
PermeaDerm AS	PermeaDerm with antiscar coating in silicone
PermeaDerm AS-2	PermeaDerm with antiscar coating in the silicone and biological layers

FDA indicates Food and Drug Administration; HMW, high molecular weight.

**Table 2:** Chemical composition*

Product/prototype	Collagen component, μg/cm^2^	Aloe, μg/cm^2^
Biobrane	68	0
PermeaDerm	10	25
PermeaDerm T	10	24

*Hydroxyproline assay (Chloramine T method by ECA) is used to estimate collagen content and the UV spectrophotometer by ECA after extraction in water at 100°C for 30 minutes is used to measure aloe content. Collagen component is high-molecular-weight type I porcine gelatin (GELITA MedellaPro gelatin) or a mixture of MedellaPro and high-molecular-weight native bovine type I collagen (in biological coating of PermeaDerm T). Aloe is Immuno-10 from Aloecorp; present in PermeaDerm and PermeaDerm T. Because of stability of biological coating of the 3-dimensional nylon/silicone structure of PermeaDerm and PermeaDerm T, it is likely that the amount reported is lower than the actual amount present.

**Table 3:** Localized effects of PermeaDerm and Biobrane

	PermeaDerm		Biobrane	
Tissue finding (range of average scores for 6 animals)	Male	Female	Male	Female
Serocellular crust (0,1)	1.0	0.9	1.0	0.9
Epidermal hyperplasia (0-4)	1.1	1.8	1.3	1.4
Reepithelialization (0-4)	0.6	1.7	0.3	1.3
Granulation tissue (0-4)	2.0	2.0	2.0	2.0
Necrotic tissue (0-4)	0.0	0.0	0.0	0.0
Inflammation (0-4)	7.0	7.0	9.7	7.6
Foreign debris (0-4)	1.3	0.8	2.9	2.5
Hemorrhage (0-4)	1.1	0.9	1.4	1.0

**Table 4:** Rat study—Wound bed adherence: PermeaDerm versus Biobrane*

Adherence	Day 7 Biobrane	Day 7 PermeaDerm	Day 14 Biobrane	Day 14 PermeaDerm
Yes	24	23	24	18
No	0	1	0	3
Partial	0	0	0	3

*The presence of gelatin/collagen increases acute adherence of the skin substitutes.

**Table 5:** Rat study—Fluid present under the skin substitute

Fluid present	Day 7 Biobrane	Day 7 PermeaDerm	Day 14 Biobrane	Day 14 PermeaDerm
Blood	22	22	7	0
Serous	4	1	0	0
Serosanguinous	2	3	0	0
Fibrinous	0	0	0	0
Purulent	0	0	0	0
None	0	0	17	24
